# Assessment of a Serum Microrna Risk Score for Colorectal Cancer among Participants of Screening Colonoscopy at Various Stages of Colorectal Carcinogenesis

**DOI:** 10.3390/cells11152462

**Published:** 2022-08-08

**Authors:** Janhavi R. Raut, Megha Bhardwaj, Tobias Niedermaier, Kaya Miah, Petra Schrotz-King, Hermann Brenner

**Affiliations:** 1 Division of Clinical Epidemiology and Aging Research, German Cancer Research Center (DKFZ), 69120 Heidelberg, Germany; 2 Medical Faculty Heidelberg, University of Heidelberg, Im Neuenheimer Feld 672, 69120 Heidelberg, Germany; 3 Division of Preventive Oncology, German Cancer Research Center (DKFZ) and National Center for Tumor Diseases (NCT), 69120 Heidelberg, Germany; 4 Division of Biostatistics, German Cancer Research Center (DKFZ), 69120 Heidelberg, Germany; 5 German Cancer Consortium (DKTK), German Cancer Research Center (DKFZ), 69120 Heidelberg, Germany

**Keywords:** miRNA, colorectal cancer, risk stratification, risk-adapted screening, blood-based

## Abstract

We recently derived and validated a serum-based microRNA risk score (miR-score) which predicted colorectal cancer (CRC) occurrence with very high accuracy within 14 years of follow-up in a large population-based cohort. Here, we aimed to assess and compare the distribution of the miR-score among participants of screening colonoscopy at various stages of colorectal carcinogenesis. MicroRNAs (miRNAs) were profiled by quantitative-real-time-polymerase-chain-reaction in the serum samples of screening colonoscopy participants with CRC (*n* = 52), advanced colorectal adenoma (AA, *n* = 100), non-advanced colorectal adenoma (NAA, *n* = 88), and participants free of colorectal neoplasms (*n* = 173). The mean values of the miR-score were compared between groups by the Mann–Whitney U test. The associations of the miR-score with risk for colorectal neoplasms were evaluated using logistic regression analyses. MicroRNA risk scores were significantly higher among participants with AA than among those with NAA (*p* = 0.027) and those with CRC (*p* = 0.014), whereas no statistically significant difference was seen between those with NAA and those with no colorectal neoplasms (*p* = 0.127). When comparing adjacent groups, miR-scores were inversely associated with CRC versus AA and positively associated with AA versus NAA [odds ratio (OR), 0.37 (95% confidence interval (CI), 0.16–0.86) and OR, 2.22 (95% CI, 1.06–4.64) for the top versus bottom tertiles, respectively]. Our results are consistent with the hypothesis that a high miR-score may be indicative of an increased CRC risk by an increased tendency of progression from non-advanced to advanced colorectal neoplasms, along with a change of the miR-patterns after CRC manifestation.

## 1. Introduction

Colorectal cancer (CRC) is the third most frequently diagnosed cancer and the second leading cause of cancer death worldwide [1]. CRC incidence and mortality have been shown to be substantially reduced by population-based screening [1,2,3,4]. While colonoscopy is regarded as the gold-standard method for early detection of CRC and CRC precursors, its invasive nature, inconvenience of bowel preparation, limited capacities, and costs [5,6,7] remain major concerns limiting its widespread use for screening. Although the fecal immunochemical test for hemoglobin has been proven to be an effective, currently available non-invasive test to screen patients who are at average risk of developing CRC, it has limited sensitivity to detect advanced colorectal adenomas or stage I CRCs [8,9]. The availability of minimally invasive risk stratification tools could potentially enhance the efficiency and cost-effectiveness of CRC screening. So far, researchers have developed risk stratification approaches to predict CRC occurrence using genetic and lifestyle-based risk models, but their predictive abilities have remained limited to date [10,11,12].

MicroRNAs (miRNAs) are a class of small noncoding RNAs that regulate gene expression and control various cellular mechanisms [13]. The dysregulation of several miRNAs has been implicated in colorectal carcinogenesis during the progression from normal mucosa to a non-advanced and advanced adenoma and an invasive tumor [14,15]. As these dysregulated miRNAs are secreted into the blood and are detectable in the serum or plasma in a highly stable form [16], circulating miRNAs measured in minimally invasive blood samples could serve as biomarkers for determining the risk of developing CRC. Previous results from our group showed that a serum-based microRNA risk score (miR-score) that integrated levels of seven miRNAs (let-7g-5p, miR-19a-3p, miR-23a-3p, miR-92a-3p, miR-144-5p, miR-21-5p, and miR-27a-3p) could predict CRC occurrence with a very high accuracy within 14 years of follow-up of a large cohort study [17]. The miR-score clearly outperformed established approaches based on environmental risk factors and genetic susceptibility loci for CRC risk prediction. While the miR-score was derived and tested for CRC risk stratification in a prospective cohort using samples collected several years before a CRC diagnosis, its distribution among average-risk and asymptomatic populations undergoing screening colonoscopy and including various stages of colorectal carcinogenesis is unknown. We aimed to assess and compare the distribution of the miR-score in a cohort of participants undergoing screening colonoscopy.

## 2. Materials and Methods

### 2.1. Study Design and Population

In Germany, from October 2002 to March 2019, a screening colonoscopy was offered from age 55 years old for both sexes. We selected samples from participants of screening colonoscopy collected in the BliTz (“Begleitende Evaluierung innovativer Testverfahren zur Darmkrebs-Früherkennung”) study. Details of the BliTz study design have been reported previously [18]. Briefly, participants of the German screening colonoscopy program, including men and women aged 55 and older, have been recruited in 20 gastroenterology practices in southern Germany since the end of the year 2005. The participants were asked to fill out a standardized questionnaire and provide blood specimens, which were processed in a central laboratory and stored in a biobank at −80 °C until analysis. Colonoscopy and histology reports were collected, and the relevant data were extracted in a standardized manner. The study has been approved by the ethics committees of the Medical Faculty Heidelberg (S-178/2005) and of the physicians’ boards of Baden-Wuerttemberg (M118-05-f), Rhineland-Palatinate [837.047.06(5145)], Hessen (MC 254/2007), and Saarland (217/13). The study adheres to the standards set by the Declaration of Helsinki, and all the study participants provided written informed consent.

**Figure 1:**
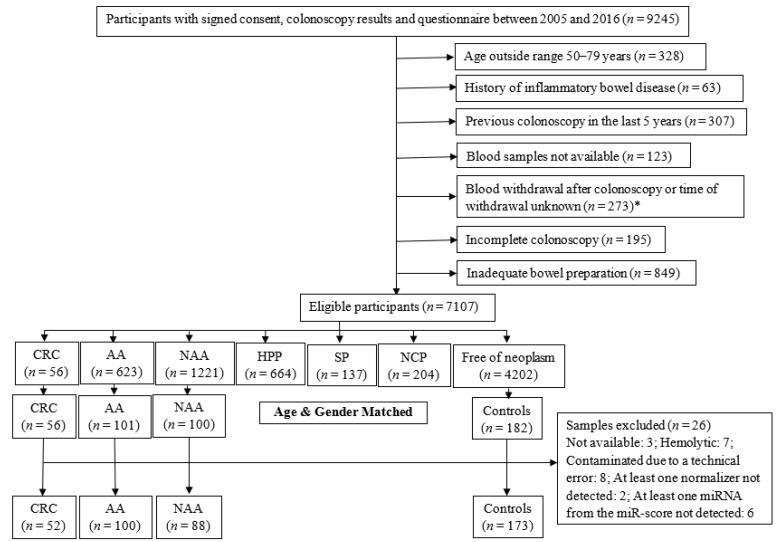
Flow diagram for selection of participants. AA, advanced colorectal adenoma; CRC, colorectal cancer; HPP, hyperplastic polyp; NAA, non-advanced colorectal adenoma; NCP, non-classified polyp; SP, serrated poly. *: The exclusion criteria for selection of CRC cases were not applicable after this point.

For the current study, we selected the serum samples of participants recruited between 2005 and 2016 (Figure 1). Eligible participants were categorized according to the most advanced finding at screening colonoscopy and included 56 CRC cases which were all selected. The participants with advanced colorectal adenoma (AA, defined as adenoma with at least 1 of the following features: size ≥ 1 cm, tubulovillous or villous components, or high-grade dysplasia, *n* = 101), non-advanced colorectal adenoma (NAA, defined as adenoma with <1 cm diameter and no tubulovillous or villous components or high-grade dysplasia, *n* = 100), and participants free of colorectal neoplasms (*n* = 173, further referred to as ‘controls’) were selected by frequency matching to the group of CRC cases based on age and sex. Next, we excluded samples with the possible influence of hemolysis or contamination on serum miRNA levels. We also excluded samples in which any one normalizer miRNA or any one target miRNA was not detected. The final selection included samples from participants with CRC (*n* = 52), AA (*n* = 100), NAA (*n* = 88), and controls (*n* = 173).

### 2.2. MiRNA Profiling by Quantitative Real-Time PCR (qPCR)

The samples were thawed on ice and centrifuged at 3000× *g* for 5 min at 4 °C. The total RNA was extracted from the samples using miRCURY™ RNA Isolation Kit (Biofluids, Qiagen, Germany) as per the manufacturer’s instructions. Two μL RNA was reversely transcribed in ten μL reactions using the miRCURY LNA RT Kit (QIAGEN). Complementary DNA (cDNA) was diluted 50× and assayed in ten μL PCR reactions according to the protocol for miRCURY LNA miRNA PCR. In a pre-analytical phase, spike-in controls UniSp2, UniSp4, and UniSp6 were added to control for RNA extraction efficiency and possible cDNA synthesis inhibitors. Hemolysis was assessed by determining the levels of miR-451 and miR-23a via qPCR. miR-451 is expressed in red blood cells, and miR-23a is relatively stable in serum and not affected by hemolysis [19,20,21]. A quantification cycle (Cq) ratio between miR-23a and miR-451 higher than 7.0 was considered indicative of sample hemolysis [22]. Corresponding samples were excluded from further analyses.

For the samples meeting the quality control criteria, seven target miRNAs (let-7g-5p, miR-19a-3p, miR-23a-3p, miR-92a-3p, miR-144-5p, miR-21-5p, and miR-27a-3p) and three normalizer miRNAs (miR-93-5p, miR-1246, and miR-223-3p) were assessed. Each miRNA was assayed once on a custom panel using miRCURY LNA SYBR Green master mix. The primers for miRNAs are listed in Supplementary Table S1. Negative controls excluding template from the reverse transcription reaction were performed and profiled similar to the samples. The amplification was performed in 384 well plates on a LightCyclerG 480 Real-Time PCR System (Roche). The amplification curves were analyzed using the Roche LC software (version 1.5.0), both for the determination of Cq (Cq was calculated as the 2nd derivative) and for melting curve analysis. The amplification efficiency was calculated using algorithms similar to the LinReg software [23]. All assays were inspected for distinct melting curves, and the melting temperature was checked to be within known specifications for the assay. Furthermore, assays within 5 Cq of the negative control or Cq > 37 were excluded from further analyses. Detectable miRNAs were those with a Cq < 40.

All of the laboratory analyses were performed blinded with respect to disease status or findings at colonoscopy.

### 2.3. Statistical Analysis

A combination of three miRNAs (miR-93-5p, miR-1246, and miR-223-3p) was identified and used to normalize qPCR data in our previous study [17]. In our current study, these normalizers exhibited good stability across the selected samples (stability value = 3.35 × 10^−3^) using the NormFinder method [24] and were identified as suitable to normalize the qPCR data. Samples with missing (raw Cq ≥ 40) values for any of the normalizers (*n* = 2) or miRNAs from the miR-score (*n* = 9) were excluded from further analyses. The data were normalized to the average Cq value of the normalizers. A miR-score was calculated for each participant using the formerly derived [17] linear predictor:

miR-score = 0.1899 + (let-7g-5p × 0.2351) + (miR-19a-3p × −0.2024) + (miR-23a-3p × 1.6595) + (miR-92a-3p × 0.4794) + (miR-144-5p × 0.2002) + (miR-21-5p × −1.6772) + (miR-27a-3p × 0.1014).

Differences in the distributions of the miR-score between the groups were assessed by the Mann–Whitney test. Tertiles of the miR-score were calculated according to the distribution of the score in the controls. Based on those tertiles, all of the participants were then categorized into three risk categories (low, medium, and high). To quantify the associations between miR-score categories and the risk for colorectal neoplasms, logistic regression analyses were performed, considering participants with low miR-score as the reference group. All models were adjusted for age and sex, and odds ratios (ORs) with corresponding 95% confidence intervals (CIs) were calculated. Additionally, using logistic regression models adjusted for age and sex, ORs per standard deviation (SD) increase in the miR-score were estimated. The performance of the miR-score for discriminating groups of participants with different colonoscopy findings was measured using the area under the receiver operating characteristic curve (AUC) values and the Brier scores. Furthermore, to assess the relationship between the miR-score and the grade of dysplasia, ORs per SD increase in the miR-score were estimated for adjacent groups, including sub-groups of AAs with and without High-grade dysplasia (i.e., CRC with advanced colorectal adenoma with High-grade dysplasia (HGDAA), HGDAA with advanced colorectal adenoma without High-grade dysplasia (WoHGDAA) and WoHGDAA with NAA).

All statistical analyses were performed with statistical software R v4.1.2 (R Core Team, 2016), together with R packages “pROC” v1.18.0 https://rdocumentation.org/packages/pROC/versions/1.18.0 and “ModelGood” v1.0.9 https://rdocumentation.org/packages/ModelGood/versions/1.0.9. For all tests, two-sided *p* values of 0.05 or less were considered to be statistically significant.

## 3. Results

### 3.1. Characteristics of the Study Population

The study population included 52, 100, and 88 participants with CRC, AA, and NAA, respectively, and 173 controls from the BliTz study (Table 1). Of the 52 CRC cases, seventeen, six, twenty-two, and seven were classified as stage I, II, III, and IV, respectively. The median age in all groups was around 65–67 years old, and males represented between 63% and 65% of participants in all groups.

### 3.2. qPCR Quality Controls

RNA extraction efficiency, monitored using UniSp2 and UniSp4, was acceptable, with raw Cq values being consistent across the dataset (UniSp2: Cq 20.31 ± 0.50, UniSp4: Cq 27.27 ± 0.54). UniSp6 was used to monitor the cDNA synthesis reactions and indicated constant efficiency of the reverse transcription step with no signs of inhibition (Cq 18.35 ± 0.22). Seven samples displayed significant hemolysis (mean Cq_mi__R-23a_ − mean Cq_miR-__451a_ > 7) and were excluded from downstream analyses.

### 3.3. Comparison of miR-Score Distributions between Groups with Various Findings at Screening Colonoscopy

Comparisons of the miR-score distributions between groups with various findings at screening colonoscopy, are presented in Table 2. Mean (SD) values of the miR-score in participants with CRC, AA, NAA and no colorectal neoplasms were −0.8 (0.7), −0.6 (0.7), −0.8 (0.7), and −0.6 (0.7), respectively. The median (interquartile range) values of the miR-score in participants with CRC, AA, NAA, and no colorectal neoplasms were −0.8 (−1.3–−0.3), −0.5 (−1.0–−0.1), −0.8 (−1.1–−0.4), and −0.6 (−1.0–−0.2), respectively. The scores were significantly higher among participants with AA than among both those with NAA (*p* = 0.027) and those with CRC (*p* = 0.014). No statistically significant difference in miR-scores was seen between participants with NAA and no colorectal neoplasms (*p* = 0.127).

When comparing adjacent groups (i.e., CRC with AA, AA with NAA, and NAA with controls), the comparison of CRC with AA group showed that having a high miR-score was inversely associated with CRC [odds ratio (OR), 0.37 (95% confidence interval (CI), 0.16–0.86) for the top versus bottom tertile]. Furthermore, for this group comparison, the OR per SD increase in the miR-score was 0.66 (95% CI, 0.47–0.94). On the other hand, when comparing AA with the NAA group, having a high miR-score was positively associated with AA [OR, 2.22 (95% CI, 1.06–4.64) for the top versus bottom tertile] and the OR per SD increase in the miR-score was 1.35 (95% CI, 1.00–1.82)]. The ROC curve analyses for discriminating participants with CRC and AA; AA and NAA; and NAA and no colorectal neoplasms resulted in AUCs (95% CIs) of 0.38 (0.28–0.47), 0.59 (0.51–0.68), and 0.44 (0.37–0.52), respectively.

Furthermore, when comparing adjacent groups, including sub-groups of AAs with and without high-grade dysplasia (Supplementary Table S2), the scores were significantly higher among participants with HGDAA than among those with CRC (*p* = 0.013). No statistically significant difference in miR-scores was seen between participants with HGDAA and WoHGDAA (*p* = 0.083) and between participants with WoHGDAA and NAA. Comparison of CRC with the HGDAA group showed that having a high miR-score was inversely associated with CRC [OR per SD increase in the miR-score was 0.32 (95% CI, 0.12–0.88)]. On the other hand, when comparing HGDAA with the WoHGDAA group, having a high miR-score was positively associated with HGDAA [OR per SD increase in the miR-score was 2.47 (95% CI, 1.03–5.90)].

## 4. Discussion

In this study, we assessed and compared the distribution of our previously reported [17] serum-based miR-score (incorporating let-7g-5p, miR-19a-3p, miR-23a-3p, miR-92a-3p, miR-144-5p, miR-21-5p and miR-27a-3p) among participants of screening colonoscopy at various stages of colorectal carcinogenesis. MiRNAs were profiled by qPCR in serum collected from participants with CRC (*n* = 52), AA (*n* = 100), NAA (*n* = 88) and controls (*n* = 173). Comparison of the miR-score distributions between groups revealed that the miR-scores were significantly higher among participants with AA than among those with NAA, suggesting a potential relationship with the transition from non-advanced to advanced adenomas. On the other hand, lower values among participants with CRC point to potential changes in miR patterns after CRC manifestation.

Numerous technologies have been developed to quantify circulating miRNA levels, but qPCR is considered to be the ‘gold standard’ owing to its high sensitivity and specificity [25,26]. Different types of qPCR platforms are commercially available to quantify miRNA levels. In our study, we used an extremely sensitive and specific approach, the miRCURY LNA miRNA PCR custom panel. The method, enabled by the use of locked nucleic acids, has been reported to exhibit better sensitivity and linearity for the detection and quantitation of low-abundance miRNAs in samples such as plasma [27]. Regarding the statistical methods for analyzing qPCR data, normalization plays a crucial role by reducing non-biological variation in the data, thereby making it easier to identify the relevant biological differences. As consensus has not been reached regarding optimal normalizer miRNAs for qPCR data analysis [28], various approaches have been used in the literature. In our previous study [17], qPCR miRNA profiling of 385 serum samples from a prospective cohort of older adults aged 50–75 years revealed that a combination of three miRNAs (miR-93-5p, miR-1246, and miR-223-3p) exhibited the highest stability across all samples. The data were normalized to the average Cq value of this normalizer combination. In the present study, the suitability of this combination was ascertained as it exhibited good stability across the BliTz study samples when evaluated using the NormFinder [24] method. However, more empirical validations are still needed to reach a consensus on robust and accurate normalizers.

Until now, most studies evaluating blood-based miRNAs in relation to CRC have focused on early detection rather than risk stratification [29,30,31,32,33,34]. Previous studies for risk stratification in CRC screening have mostly used risk scores based on environmental risk factors and genetic susceptibility loci to determine CRC risk [10,11,12]. A former study [35] from our research group assessed associations of a polygenic risk score (PRS) and a healthy lifestyle score (HLS) with the presence of non-advanced adenomas and advanced neoplasms (the combined group including AA and CRC) in screening program participants from the BliTz cohort. Both the PRS and the HLS showed positive individual associations with advanced neoplasms versus non-advanced adenomas [OR, 1.65 (95% CI, 1.23–2.22) and OR, 1.38 (95% CI, 1.01–1.89) for the top versus bottom tertile, *p*_trend_ 0.003 and 0.007, respectively]. A combination of PRS and HLS was also positively associated with advanced neoplasms versus non-advanced adenomas, considering the lowest risk group (low genetic risk and favorable lifestyle) as the reference group [OR, 2.26 (95% CI, 1.31–3.92)]. Our study evaluated the use of a miRNA-based score as a risk stratification tool in screening program participants from the same BliTz cohort. The miR-score was positively associated with advanced versus non-advanced adenomas [OR, 2.22 (95% CI, 1.06–4.64) for the top versus bottom tertile] but inversely associated with CRC versus advanced adenomas [OR, 0.37 (95% CI, 0.16–0.86) for the top versus bottom tertile]. Our findings are largely consistent with previous literature indicating a high miR-score being associated with increased CRC risk by an increased tendency of progression from non-advanced to advanced colorectal neoplasms, along with changing miRNA patterns in the years prior to CRC diagnosis [17,36].

A previous study [36] comparing the miRNA levels between samples of CRC cases collected years before diagnosis versus samples collected at the time of diagnosis revealed major changes among most of the cases that seemed to occur mainly in the three years prior to diagnosis. Another study [37] investigated changes in serum miRNA profiles over time in samples from ten patients with colon cancer collected at three time points prior to diagnosis (up to 32 years prior to diagnosis with a median of 5 years interval between time points), one time-point after diagnosis, and from individually matched controls. When comparing samples collected at the three pre-diagnostic time points with samples from matched controls, no clearly dysregulated miRNA signature was identified at the first and second time points. However, at time point three closest to diagnosis, a group of down-regulated miRNAs was identified. Furthermore, the comparison between post-diagnostic samples and the combined controls revealed a prominent group of up-regulated miRNAs, indicating the presence of time-specific miRNA profiles during the course of colon cancer development. In this context, the results of our previous study [17], using samples from incident CRC cases (collected in median 6.8 years prior to diagnosis) and controls revealed dysregulations of some miRNAs that were contradictory to studies in the literature using samples from diagnosed CRC cases and controls. Considering that a majority of incident CRC cases probably developed in participants who had AA at the time of blood sampling or who developed AA from NAA after blood sampling and later developed CRC, having a high miR-score was associated with a significantly increased risk of developing CRC in the future. In our current study, the observation that the miR-scores were significantly lower among participants with CRC than among participants with AA reinforces the notion that miRNA profiles continue to alter as the development of cancer progresses [38], with major changes mostly occurring in the years closer to cancer diagnosis [36,37].

The strengths of our study include the assessment of circulating miRNA profiles in a large average-risk and asymptomatic population attending screening colonoscopy, which is an ideal target population for assessing novel biomarkers of CRC risk. Moreover, our population included well-characterized groups of participants corresponding to the sequential stages of CRC development, rendering a thorough comparison of the distribution and discriminatory ability of the miR-score in this population. However, our study also has some important limitations. In particular, despite the large size of the BliTz cohort, this study was based on a limited number of CRC cases, a feature that is common for screening settings. Furthermore, serum samples were evaluated at only one time point, which prohibited the assessment of intra-individual variability and dynamic changes in miRNA profiles before and after diagnosis. Thus, the results from our previous [17] and the current study, suggesting consistently-altering serum miRNA profiles during CRC progression, remain preliminary, and should be investigated further in future large-scale studies that directly compare miRNA levels in samples collected at different time points from the same set of participants.

In conclusion, the results of this analysis are consistent with the hypothesis that a high miR-score may be indicative of an increased CRC risk by an increased tendency of progression from non-advanced to advanced colorectal adenomas, along with a change of the miR-patterns after CRC manifestation. We found that the miR-scores are significantly different between various stages of colorectal carcinogenesis, which may affect the potential clinical application for risk-adapted CRC screening. Future large-scale studies investigating alterations in miRNAs patterns over time and in relation to colorectal carcinogenesis are needed to more accurately define their potential use for CRC risk stratification.

## Figures and Tables

**Table 1:** Characteristics of the Study Population.

Characteristics	CRC Cases (*n* = 52)	AA Cases (*n* = 100)	NAA Cases (*n* = 88)	Controls (*n* = 173)
Age (Years)				
Mean (SD)	66.3 (6.1)	65.4 (6.6)	65.2 (6.1)	65.7 (6.7)
Median (Interquartile Range)	67 (62–71)	65 (60–71)	65 (61–70)	66 (60–71)
Sex (%)				
Male	33 (63.5)	65 (65.0)	55 (62.5)	109 (63.0)
Female	19 (36.5)	35 (35.0)	33 (37.5)	64 (37.0)
TNM stage—counts (%)				
Stage I	17 (32.7)	-	-	-
Stage II	6 (11.5)	-	-	-
Stage III	22 (42.3)	-	-	-
Stage IV	7 (13.5)	-	-	-

Abbreviations: AA, advanced colorectal adenoma; CRC, colorectal cancer; NAA, non-advanced colorectal adenoma; *n*, number; SD, standard deviation; TNM, Tumour Nodes Metastasis classification.

**Table 2:** Comparison of miR-Score Distributions Between Groups with Various Findings at Screening Colonoscopy.

miR-Score		Comparison CRC–AA		Comparison AA–NAA		Comparison NAA–No Neoplasm	
	CRC (*n* = 52)		AA (*n* = 100)		NAA (*n* = 88)		No neoplasm (*n* = 173)
Mean (SD)	−0.8 (0.7)		−0.6 (0.7)		−0.8 (0.7)		−0.6 (0.7)
Median (Range)	−0.8 (−2.6–0.7)		−0.5 (−2.4–1.2)		−0.8 (−2.6–0.9)		−0.6 (−3.3–3.0)
*p*-Value ^a^		**0.014**		**0.027**		0.127	
							
miR-Score Category ^b^		OR (95% CI) ^c^		OR (95% CI) ^c^		OR (95% CI) ^c^	
Low	24 (46.2)	Ref	28 (28.0)	Ref	32 (36.4)	Ref	58 (33.5)
Medium	15 (28.8)	0.54 (0.23−1.22)	32 (32.0)	1.05 (0.52−2.12)	35 (39.8)	1.07 (0.58−1.98)	57 (32.9)
High	13 (25.0)	**0.37 (0.16−0.86)**	40 (40.0)	**2.22 (1.06−4.64)**	21 (23.9)	0.63 (0.32−1.24)	58 (33.5)
OR per SD Increase		**0.66 (0.47−0.94)**		**1.35 (1.00−1.82)**		0.79 (0.61−1.04)	
							
AUC	0.38 (0.28−0.47)			0.59 (0.51−0.68)		0.44 (0.37−0.52)	
Brier Score	0.269			0.280		0.257	

^a^ Assessed by Mann–Whitney test. ^b^ Tertiles of risk score among participants with no colorectal neoplasms (controls). ^c^ Adjusted for age and sex. Bold values indicate statistically significant results. Abbreviations: AA, advanced colorectal adenoma; AUC, area under the receiver operating characteristic curve; CI, confidence interval; CRC, colorectal cancer; miR-score, microRNA risk score; *n*, number; NAA, non-advanced colorectal adenoma; OR, odds ratio; Ref., reference category; SD, standard deviation.

## Data Availability

All data that support the findings of this study are available on reasonable request from the corresponding author (H.B.). The data are not publicly available due to them containing information that could compromise research participant privacy/consent.

## Supplementary Materials

The following are available online at https://www.mdpi.com/article/10.3390/cells11152462/s1, Table S1: Information on the quantitative real-time polymerase chain reaction primers (QIAGEN); Table S2: Comparison of miR-score distributions between groups with various findings at screening colonoscopy including sub-groups of advanced colorectal adenomas with and without high-grade dysplasia.

## Abbreviations

AA	advanced colorectal adenoma
AUC	area under the receiver operating characteristic curve
BliTz	Begleitende Evaluierung innovativer Testverfahren zur Darmkrebs-Früherkennung
cDNA	complementary DNA
CI	confidence interval
Cq	quantification cycle
CRC	colorectal cancer
HPP	hyperplastic polyp
HLS	healthy lifestyle score
miRNA	microRNA
miR-score	microRNA risk score
*n*	number
NAA	non-advanced colorectal adenoma
NCP	non-classified polyp
OR	odds ratio
PRS	polygenic risk score
qPCR	quantitative real-time polymerase chain reaction
Ref.	reference category
SD	standard deviation
SP	serrated poly
TNM	Tumour Nodes Metastasis classification
