# Supplementary material for: Assessment of a Serum Microrna Risk Score for Colorectal Cancer among Participants of Screening Colonoscopy at Various Stages of Colorectal Carcinogenesis
Source: Cells. 2022 Aug 8;11(15):2462. doi: 10.3390/cells11152462 (PMC9367813; doi:10.3390/cells11152462)
Supplement: Supplementary file 1 [file cells-11-02462-s001.zip › cells-1809007-supplementary.pdf]

Supplementary materials for:

# Assessment of a serum microRNA risk score for colorectal cancer among participants of screening colonoscopy at various stages of colorectal carcinogenesis

Janhavi R. Raut[1,2,3], Megha Bhardwaj[1], Tobias Niedermaier[1], Kaya Miah[4], Petra Schrotz-King[3] and Hermann Brenner[1,3,5]

**Supplementary Table S1. Information on the quantitative real-time polymerase chain reaction primers (QIAGEN).**

| | microRNA | Accession | Catalog number | Sequence |
|---|---|---|---|---|
| 1 | let-7g-5p | MIMAT0000414 | YP00204565 | UGAGGUAGUAGUUUGUACAGUU |
| 2 | miR-19a-3p | MIMAT0000073 | YP00205862 | UGUGCAAAUCUAUGCAAAACUGA |
| 3 | miR-23a-3p | MIMAT0000078 | YP00204772 | AUCACAUUGCCAGGGAUUUCC |
| 4 | miR-92a-3p | MIMAT0000092 | YP00204258 | UAUUGCACUUGUCCCGGCCUGU |
| 5 | miR-144-5p | MIMAT0004600 | YP00204670 | GGAUAUCAUCAUAUACUGUAAG |
| 6 | miR-21-5p | MIMAT0000076 | YP00204230 | UAGCUUAUCAGACUGAUGUUGA |
| 7 | miR-27a-3p | MIMAT0000084 | YP00206038 | UUCACAGUGGCUAAGUUCCGC |
| 8 | miR-93-5p | MIMAT0000093 | YP00204715 | CAAAGUGCUGUUCGUGCAGGUAG |
| 9 | miR-1246 | MIMAT0005898 | YP00205630 | AAUGGAUUUUUGGAGCAGG |
| 10 | miR-223-3p | MIMAT0000280 | YP00205986 | UGUCAGUUUGUCAAAUACCCCA |

**Supplementary Table S2. Comparison of miR-score distributions between groups with various findings at screening colonoscopy including sub-groups of advanced colorectal adenomas with and without high-grade dysplasia.**

| miR-score | | Comparison CRC–HGDAA | | Comparison HGDAA–WoHGDAA | | Comparison WoHGDAA–NAA | |
|---|---|---|---|---|---|---|---|
| | CRC (n = 52) | | HGDAA (n = 9) | | WoHGDAA (n = 91) | | NAA (n = 88) |
| Mean (SD) | −0.8 (0.7) | | −0.2 (0.7) | | −0.6 (0.7) | | −0.8 (0.7) |
| Median (range) | −0.8 (−2.6–0.7) | | −0.1 (−1.3–1.2) | | −0.6 (−2.4–0.6) | | −0.8 (−2.6–0.9) |
| *p*-value[a] | | **0.013** | | 0.083 | | 0.068 | |
| | | | | | | | |
| | | OR (95% CI)[b] | | OR (95% CI)[b] | | OR (95% CI)[b] | |
| OR per SD increase | | **0.32 (0.12–0.88)** | | **2.47 (1.03– 5.90)** | | 1.27 (0.94–1.72) | |

[a] Assessed by Mann-Whitney test. [b] Adjusted for age and sex. Bold values indicate statistically significant results. Abbreviations: CI, confidence interval; CRC, colorectal cancer; HGDAA, advanced colorectal adenoma with high-grade dysplasia; miR-score, microRNA risk score; n, number; NAA, non-advanced colorectal adenoma; OR, odds ratio; SD, standard deviation; WoHGDAA, advanced colorectal adenoma without high-grade dysplasia.
